# Long-term effect of repeated application of pig slurry digestate on microbial communities in arable soils

**DOI:** 10.1016/j.heliyon.2024.e41117

**Published:** 2024-12-10

**Authors:** Daniela Mora-Salguero, Lionel Ranjard, Thierry Morvan, Samuel Dequiedt, Vincent Jean-Baptiste, Sophie Sadet-Bourgeteau

**Affiliations:** a Agroécologie, French National Institute for Agriculture, Food, and Environment (INRAE), Institut Agro, Univ. Bourgogne, Univ. Bourgogne Franche-Comté, Dijon, France; b French National Institute for Agriculture, Food, and Environment (INRAE), UMR Sol Agro el hydrosystème Spatialisation, Rennes, France; c Gaz Réseau de France (GRDF), Paris, France; d Institut Agro Dijon, France

**Keywords:** Anaerobic digestion, Soil microbial communities, High-throughput sequencing, Long-term fertilization, Agricultural practices

## Abstract

Anaerobic digestion represents an opportunity for converting organic waste (OW) into valuable products: renewable energy (biogas) and a fertilizer (digestate). However, the long-term effects of digestates on soil biota, especially microorganisms, need to be better documented to understand the impact of digestate on soil ecosystem functioning and resilience. This study assessed the cumulative effect of repeated pig slurry digestate applications on soil microbial communities over a decade, using an in-situ approach to compare digested feedstock with undigested feedstock and other fertilization treatments. Conducted from 2012 to 2022 at an experimental field site in France, the study involved plots with identical agricultural soil management practices, differing only in fertilization treatments: mineral fertilizer, three different OW (cattle manure, pig slurry, pig slurry digestate), and a control with no organic or mineral fertilizer input. Changes in soil microbial communities were analyzed through molecular microbial biomass and diversity assessments using high-throughput sequencing targeting 16S and 18S ribosomal RNA genes. DNA extraction and molecular analyses were performed on soil samples collected at the start of the trial in 2012 and subsequently in 2017 and 2022. The long-term effects of annual digestate application over a decade include a higher soil microbial diversity in digestate-treated plots than in pig slurry-treated plots, and changes in the soil's microbial community structure and taxonomic composition resembling those observed with mineral fertilizer. Differential abundance analysis at the phylum level revealed few significant differences between digestate- and mineral fertilizer-treated plots for both prokaryotic and fungal communities. Only plots amended with cattle manure exhibited higher soil organic carbon content. Agricultural practices, along with climatic and environmental fluctuations, can significantly influence the response of soil microbial communities, thereby buffering the effects of fertilization treatments. Further research is needed to better understand the effects on soil microbial communities, considering the interactions between repeated digestate applications, different pedological contexts, and agricultural practices.

## 1 Introduction

Soils are complex physicochemical matrices with a porous structure, a huge surface area, and an extremely variable supply of organic materials, nutrients, and water, providing a range of habitats for a multitude of organisms [1]. They make up one of the most diverse habitats on Earth. Nowhere in nature are species so densely clustered as in soil communities [2]. Microbial communities account for a large part of this biodiversity. Estimates of soil microbial diversity range from thousands to a million microbial species in just a few grams of soil [3]. Soil microbial communities (Archaea, Bacteria, Fungi) play a crucial role in ecosystem functioning and resilience, particularly in biogeochemical cycles, organic matter mineralization, plant growth and productivity, soil structure maintenance, and soil pollutant reduction [[4], [5], [6], [7], [8]]. Soil microbial communities are good indicators for monitoring soil quality and serve as early bioindicators of disturbances due to their essential role in soil functions and their high sensitivity to changes in soil conditions, combined with their short generation time [9]. Recent advances in molecular biology have provided access to a large part of the soil microbial diversity, e.g., taxonomic richness or community composition [10]. Moreover, several studies have reported that soil microorganisms serve as early and robust indicators of the impact of agricultural practices that threaten crop production sustainability, due to their small size, rapid generation time, and sensitivity to soil disturbances [9,11,12].

Soils are fundamental resources for agricultural production and food security. As providers of more than 95 % of global food, they depend on intensive agricultural practices [13]. Sustainable agriculture should satisfy global needs, i.e., not only food supply but also a range of other issues involving energy use, nutrient recycling, and the effects on adjacent ecosystems, including the impact on water bodies and climate change [14]. Aiming for an agroecological transition, the biogas sector can represent a sustainable opportunity by developing (i) the production of renewable energies such as electricity, or biomethane, and (ii) the management of organic waste (OW) [15]. Biogas is produced from the anaerobic digestion of organic matter. A wide variety of feedstock can be used (crop residues, animal manure, the organic fraction of municipal and industrial solid waste, or wastewater sludge) [16]. This process converts biowaste into two economically useful products: renewable energy (biogas) and a fertilizer (digestate) [17]. However, reports on the impacts of digestates on the soil microbiota are scarce, and rather contradictory, as reviewed by Karimi et al. [15].

Most of the studies conducted to assess the impact of digestates on soil microbiota have been carried out under laboratory conditions often poorly linked to real-life agronomic conditions [15]. For instance, some studies in short- (≤ 1 year) and mid- (≤ 5 years) term on field experiments reported positive or neutral effects after digestate application on soil organic carbon content (SOC) and some microbial parameters (e.g., microbial biomass and diversity) [[18], [19], [20], [21], [22]]. Interestingly, Coelho et al. [22] observed that microbial communities from different liquid digestates failed to establish in the soil after two years of repeated applications. However, studies conducted on long-term fertilization (≥5 years; organic and/or mineral fertilization) have reported more persistent impacts on soil characteristics [23,24], plant growth [25], and microbial diversity and activity [[26], [27], [28]], suggesting that repeated digestate application over five years could have a lasting effect on soil microbial communities To our knowledge, no studies have assessed the long-term dynamics of soil microbial communities following repeated digestate applications. Therefore, no objective and robust conclusions can be drawn regarding the impact of digestate on soil microbial biodiversity, particularly in a real field context, with repeated applications over the long term [15,29].

The field experiment “Effluents d'Elevage et Environnement” (EFELE; Rennes, France) was set up in 2012. It aims to comprehensively understand the long-term effects of repeated fertilization with different types of organic wastes (OW) on the soil quality and functioning. The experiment involved different fertilization practices, including mineral and organic inputs (cattle manure, pig slurry and pig slurry digestate). The present study aimed to assess the response of soil microbial communities (Archaea, Bacteria, Fungi) to 10 years of mineral and OW fertilization regimes by molecular DNA-based tools targeting the soil microbial biomass, soil microbial diversity, and soil microbial community structure. To our knowledge, no report had as yet employed an *in-situ* approach to monitor the dynamic effect of the repeated application of a digestate over a decade on soil microbial parameters. We hypothesized that (i) long-term historical and repeated digestate applications would strongly influence the edaphic soil properties, and in turn, affect the soil microbial parameters; and (ii) lasting modifications would only occur after several years of repeated applications. We assessed the long-term impact of historical and repeated fertilization practices on soil microbial communities, using digestate as an organic fertilizer. We also evaluated whether the digestate feedstock had a similar impact to that of the same undigested feedstock and other commonly used types of fertilizers.

## 2 Materials and methods

### 2.1 Experimental site, soil sampling strategy, and soil chemical analysis

The study was carried out at the EFELE experimental site (Le Rheu, France; 48°06′07″N, 1°47′44″W) of the SOERE-PRO^1^ network, designed for long-term studies on the evolution of agrosystems after repeated applications of OW (see Refs. [30,31] for a complete description). The soil was classified as a Luvisol-Redoxisol derived from aeolian silt deposited on schist material [32]. The experiment was set up in 2012. The topsoil horizon (0–25 cm soil depth) had the following properties at the beginning of the trial: clay 14.2 %; silt 70.8 %; sand 15 %; total C 11.03 g kg^−1^; total N 1.14 g kg^−1^; C/N ratio 9.62; pH 6.10. The climate was mild temperate oceanic, with 711 mm year^−1^ mean annual rainfall and 11.2 °C mean annual temperature.

**Fig. 1:**
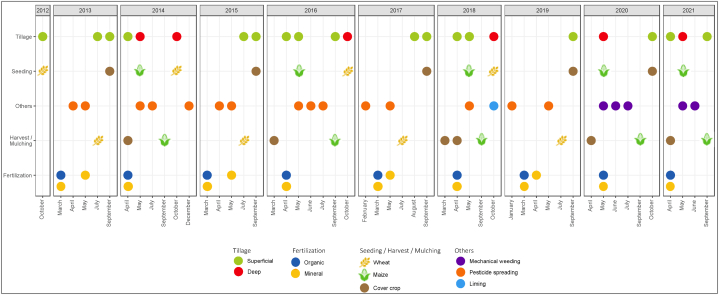
Agricultural practices at the EFELE from 2012 to 2021 on all treatments, including crop succession, fertilization, and soil management activities. The experiment was based on a winter wheat (*Triticum aestivum* L.) and maize (*Zea mays* L.) crop rotation, with a cover crop of white mustard (*Sinapis alba*). Wheat and maize were harvested, whereas mustard was mulched. Tillage was classified depending on soil depth (superficial <10 cm; deep >10 cm). Fertilization treatments: MIN, mineral fertilizer; ON, control that received no organic or mineral N input; three different OW: CM, cattle manure; PS, pig slurry; PS-DIG, pig slurry digestate. “Others” management practices grouped: mechanical weeding, pesticide spreading, and liming. The liming rate was 370 kg ha^−1^(granules containing 54.5 % CaO and 0.5 % MgO). Soil samples were collected in March every year from 2012 to 2022.

The experiment was based on a winter wheat (*Triticum aestivum* L.) and maize (*Zea mays* L.) crop rotation, with a catch crop of white mustard (*Sinapis alba*) (Fig. 1). The crop residues (including aboveground residues) were not returned to the soil. Before the start of the experiment in 2010 and 2011, maize was grown as a homogenization crop. The soil management activities performed from 2012 to 2022 are presented in Fig. 1. The fertilization treatments included a mineral N fertilizer (MIN) in the form of ammonium nitrate (comprising 50 % nitrate and 50 % ammonium), a control (ON) that did not receive any organic or mineral nitrogen input (but fertilized with P and K), and three different OW: cattle manure (CM), pig slurry (PS), and the digestate obtained after anaerobic digestion of the same pig slurry used in the study (PS-DIG). The design of the trial was a complete randomized block where each treatment had 4 replicates, and each plot had a surface area of 109 m^2^ (12.5 m × 8.7 m). MIN, PS, and DIG-PS were applied once a year in early spring (late March to mid-April), and CM every two years. The mean characteristics of the OW applied from 2012 to 2022 are given in Table 1. The index of residual organic carbon (I_ROC_) represents the proportion of organic matter that may be incorporated into the soil organic matter following OW application [33]. The mean rate of MIN applied from 2012 to 2021 represented 110.9 kg N ha^−1^ yr^−1^, 52.5 kg P_2_O_5_ ha^−1^ yr^−1^, and 78 kg K_2_O ha^−1^ yr^−1^. The detailed amounts of OW and mineral fertilizer applied *per* year are presented in Table S 1, Figure S 2, and Figure S 3. The application rates for the liquids OW (PS-DIG and PS) were determined according to their N fertilizer value, aligning with the regulations of the EU Nitrates Directive (91/676/EEC [34]). The CM inputs (solid OW) were reasoned based on the first limiting element, phosphorus, aiming not to exceed the threshold of an annual input of 100 kg P_2_O_5_ ha^−1^ yr^−1^. The application rate of MIN was calculated using the mineral N balance method recommended in France [35]. Except for the ON plots, all plots, regardless of the treatments, showed similar yields throughout the years (18.65 t DM/ha for maize silage; 8.23 t DM/ha for wheat straw and 82.06 quintal/ha for wheat grain).

**Table 1:** Mean characteristics of the organic wastes (OW) applied in the EFELE plots from 2012 to 2021.

Treatment	Applied quantity	Dry matter	Organic C	Total N	Organic N	I_ROC_	C/N	pH (water)
	t FM ha^−1^ yr^−1^	%	kg FM ha^−1^ yr^−1^	kg FM ha^−1^ yr^−1^	kg FM ha^−1^ yr^−1^	% Organic C		
**CM**	25a (0.0)	26.2a (3.6)	4692.5a (818.8)	313.3a (60.9)	248.1a (59.2)	58.8a (6.6)	15.2a (2.4)	9.4a (0.4)
**PS**	31.4a (8.4)	7.2b (2.5)	844.5b (397.5)	160.6b (47.1)	41.6b (17.7)	49.3a (10.4)	5.2b (1.3)	8.5a (1.0)
**PS-DIG**	32.9a (8.0)	5.8b (1.7)	645.3b (217.3)	159.5b (34.8)	36.8b (8.3)	54.7a (10)	4.0b (0.9)	9.0a (1.0)

Fresh matter (FM). Values are means and letters denote the significant effect between treatments (p < 0.05) based on Tukey's HSD test p-value adjusted by the BH method. The standard error of the means is indicated in parentheses. CM, cattle manure; PS, pig slurry; PS-DIG, pig slurry digestate.

Soil samples were collected in early March (about 11 months after the latest fertilization treatment) every year from 2012 to 2022. The last soil sampling was in March 2022. Each sample was composed of 8 soil cores taken from the 0–25 cm horizon at random locations in each plot, and mixed and homogenized by 4 mm mesh sieving to remove above-ground plant material, other plant debris, roots, and stones. The sieved soil was freeze-dried and stored at −40 °C at the GenoSol soil conservatory (INRAE, Dijon, France).^2^ DNA extraction and molecular analyses were conducted in 2022 on soil samples collected at the beginning of the trial in 2012, as well as 5 and 10 years later (in 2017 and 2022, respectively). This led to a total of 60 soil samples (5 treatments × 4 replicates × 3 years). A portion of the same soils sampled in 2012, 2016, and 2021 was air-dried for physicochemical analysis to determine soil particle size distribution, pH, SOC, soil total nitrogen, the soil C/N ratio, total phosphorus, and the cation exchange capacity (CEC). These analyses were performed at the INRAE Soil Laboratory Analysis.^3^ The soil physicochemical data are given in Table 2.

**Table 2:** Soil physicochemical parameters of the EFELE plots according to the fertilization treatment and the sampling time.

Treatment	Organic carbon	Total N	P_2_O_5_ (Olsen)	C:N	pH (water)	CEC
	g kg^−1^	g kg^−1^	g kg^−1^			cmol + kg^−1^
**2012**						
**CM**	10.77a (0.574)	1.13a (0.05)	0.18a (0.02)	9.53a (0.05)	6.14a (0.05)	6.17a (0.20)
**MIN**	11.45a (0.532)	1.19a (0.06)	0.18a (0.03)	9.61 ab (0.08)	5.97a (0.24)	6.10a (0.65)
**ON**	11.16a (0.935)	1.15a (0.09)	0.19a (0.02)	9.72b (0.11)	6.14a (0.27)	6.36a (0.55)
**PS**	10.95a (0.443)	1.13a (0.04)	0.19a (0.02)	9.62 ab (0.08)	6.16a (0.21)	6.44a (0.58)
**PS-DIG**	10.51a (0.878)	1.10a (0.09)	0.17a (0.01)	9.57 ab (0.05)	5.99a (0.25)	5.71a (0.41)
**2016**						
**CM**	11.12a (0.36)	1.19a (0.02)	0.14a (0.02)	9.37a (0.09)	6.16a (0.11)	6.63a (0.47)
**MIN**	10.8a (0.53)	1.16a (0.06)	0.13a (0.02)	9.28a (0.03)	5.91a (0.20)	6.01a (0.63)
**ON**	10.42a (0.84)	1.12a (0.07)	0.14a (0.02)	9.26a (0.14)	6.13a (0.19)	6.29a (0.48)
**PS**	10.95a (0.64)	1.17a (0.06)	0.15a (0.02)	9.38a (0.08)	6.26a (0.14)	6.62a (0.50)
**PS-DIG**	10.45a (0.65)	1.12a (0.07)	0.14a (0.01)	9.29a (0.02)	6.07a (0.16)	5.92a (0.26)
**2021**						
**CM**	11.27b (0.39)	1.25b (0.05)	0.14a (0.01)	9.03a (0.09)	6.36a (0.14)	6.55a (0.28)
**MIN**	9.89a (0.23)	1.11a (0.04)	0.14a (0.02)	8.89a (0.17)	5.85b (0.16)	5.80a (0.45)
**ON**	9.61a (0.39)	1.08a (0.05)	0.17a (0.03)	8.93a (0.09)	6.17 ab (0.31)	5.67a (1.21)
**PS**	10.14a (0.32)	1.15 ab (0.04)	0.15a (0.01)	8.83a (0.07)	6.14 ab (0.13)	6.38a (0.46)
**PS-DIG**	9.79a (0.53)	1.09a (0.06)	0.14a (0.01)	8.94a (0.04)	6.06 ab (0.22)	5.82a (0.28)

Data pertains to air-dried soil. Values are means and letters denote the significant effect of treatment for each sampling time (p < 0.05) based on Tukey's HSD test p-value adjusted by the BH method. The standard error of the means is indicated in parentheses. CM, cattle manure; PS, pig slurry; MIN, mineral fertilizer; ON, no fertilization; PS-DIG, pig slurry digestate.

### 2.2 DNA extraction and purification

Total DNA was extracted from 1 g (dry weight) of soil using a single procedure standardized by the GenoSol platform (INRAE, Dijon, France)^2^ [36]. This protocol is based on three main steps: (i) microbial cell lysis by physical and chemical action; (ii) deproteinization; and (iii) alcohol precipitation and washing of the extracted nucleic acids. The DNA concentrations of the crude extracts were determined by electrophoresis in 1 % agarose gel stained with ethidium bromide, using calf thymus DNA as a standard curve. Quantified crude DNA was used as an estimate of the soil molecular microbial biomass [37]. In order to separate residual impurities – particularly humic substances – 100 μL of crude DNA was purified using a Nucleospin® Soil kit (Macherey-Nagel GmbH & Co. KG, Düren, Germany). The concentrations of purified DNA were finally measured using a Quantifluor staining kit (Promega, Madison, Wisconsin, USA), according to the manufacturer's instructions.

### 2.3 High throughput sequencing of 16S and 18S rRNA gene sequences

Prokaryotic (bacterial-archaeal) diversity was estimated from each DNA sample by metabarcoding of the 16S rRNA gene following the method described by [38]. A 440-base fragment targeting the V3 to V4 regions was first amplified with the corresponding primers F479 (5′CAG CMG CYG CNG TAA NAC3′) and R888 (5′CCG YCA ATT CMT TTR AGT3′). Fungal diversity was estimated by metabarcoding of the 18S rRNA gene. A 350-base fragment targeting the V7 to V8 regions was first amplified with the corresponding primers FR1 (5′ANC CAT TCA ATC GGT ANT3′) and FF390 (5′CGA TAA CGA ACG AGA CCT3′) according to [39].

For each sample, PCR amplifications were performed with 5 ng of DNA in a total reaction mixture volume of 25 μL. The thermal profile of the prokaryotic PCR was as follow: initial denaturation at 94 °C for 2 min, 35 cycles of denaturation at 94 °C for 30 s, annealing at 52 °C for 30 s, and extension at 72 °C for 1 min, followed by a final extension period at 72 °C for 7 min. The thermal profile of the fungal PCR was as follow: initial denaturation at 94 °C for 3 min, 35 cycles of denaturation at 94 °C for 30 s, annealing at 52 °C for 1 min, and extension at 72 °C for 1 min, followed by a final extension period at 72 °C for 5 min. All PCR products were purified with an Agencourt® AMPure XP kit (Beckman Coulter, Brea, California, USA) and quantified with a Quantifluor staining kit (Promega, Madison, Wisconsin, USA).

A second PCR was run on the purified PCR products (7.5 ng of DNA for bacteria and archaea, 5 ng for fungi), using 10-base-pair multiplex identifiers (MIDs) added at the 5′ and 3’ ends of the primers for subsequent sample identification. The thermal profile of the second PCR for the preparation of bacterial and archaeal rDNA amplicon libraries was the same as previously described, but with only 7 cycles. The same procedure was followed for the preparation of fungal rDNA amplicon libraries (same thermal profile as in the first PCR, but only 7 cycles). Moreover, the denaturation step lasted 1 only min. All PCR products were purified using a MinElute PCR purification kit (Qiagen NV) and quantified with a Quantifluor staining kit (Promega, Madison, Wisconsin, USA). For all libraries, the samples were pooled at equimolar concentrations and purified using an Agencourt® AMPure XP kit (Beckman Coulter, Brea, California, USA) to remove excess nucleotides, salts, and enzymes. Finally, sequencing was carried out with a NovaSeq Illumina instrument (Illumina Inc. San Diego, California, USA) producing 250-bp paired reads.

### 2.4 Bioinformatic analysis of 16S and 18S rRNA gene sequences

Bioinformatic analyses were performed using the BIOCOM-PIPE pipeline [40]. First, all 16S and 18S raw reads were sorted according to the MID sequences. Data preprocessing included initial trimming of raw reads using PRINSEQ and merging of paired-end reads using FLASH. The low-quality reads were discarded based on their minimum length, number of ambiguities (Ns), and primer sequences. Then, the reads were dereplicated to save computing time in the subsequent steps of the pipeline (i.e., clustering of strictly identical sequences). The dereplicated reads were aligned using Infernal alignment tool [41], and clustered into operational taxonomic units (OTUs) with a similarity threshold of 95 %. A filtering step was carried out to remove chimeras based on the quality of their taxonomic alignments [40]. Finally, the retained reads were homogenized by random selection of 10,000 reads to compare the datasets efficiently and avoid biased community comparisons. As observed in previous studies, if sequencing depth is sufficient, such a homogenization step will produce robust results when describing patterns in α- and β-diversity [42,43]. The retained high-quality reads were used for (i) the re-clustering step using ReClustOR to improve OTU consistency based on a reference OTU database [44] taken from the RMQS project (French Soil Quality Monitoring Network) [45]; (ii) taxonomy-independent analyses to calculate diversity indexes by Hill numbers (richness, Shannon, Inverse Simpson) using the OTU dataset; and (iii) taxonomy-based analyses by similarity approaches against curated reference databases from SILVA r132 [46]. The raw datasets are available in the EBI database system under project accession number PRJEB71034.

### 2.5 Statistical analyses

All statistical analyses were performed with R studio (RStudio, Version 2022.12.0 + 353, Posit Software, PBC formerly RStudio, Boston, Massachusetts, USA) using the free statistics software R (version 4.2.2, 2022-10-31). OW characteristics, soil physicochemical parameters, soil molecular microbial biomass, alpha diversity index, and the relative abundance of soil prokaryotic and fungal *phyla* were processed by a two-way mixed analysis of variance (ANOVA). The model included the plot identity as a random factor to account for repeated measures within each plot, with ‘time’ as the within-subject factor and ‘treatment’ as the between-subject factor. A Tukey's HSD *post hoc* test with the Benjamini & Hochberg (BH) correction method was applied to adjust the p-values.

Alpha diversity was analyzed using Hill numbers generated by the vegan package [47]. Hill numbers allow interpreting alpha diversity linearly by progressively considering the abundances of rare and dominant OTUs using the scaling parameter q (order of diversity) [48]. Therefore, q = 0 represents richness, q = 1 corresponds to the exponential of Shannon entropy, weighing OTUs by their frequency without disproportionate favoring either rare or abundant ones, and q = 2 represents the inverse of Simpson index, where the abundant OTUs are overweighed [48]. Similarities in the compositions of the prokaryotic and fungal communities among different fertilization treatments over time were evaluated by an analysis of similarities (ANOSIM) and an analysis of multivariate homogeneity of group dispersions (beta-dispersion), using 999 permutations and a significance threshold *p* < 0.05, based on the robust Aitchison dissimilarity distance [49].

To visualize the distribution patterns of microbial communities for each fertilization treatment over time, the non-metric multidimensional scaling (NMDS) approach was employed based on the robust Aitchison dissimilarity distance [49], using the *metaMDS* function from the *vegan* package [47]. The significance of the differences between treatments and over time was assessed using a non-parametric permutational multivariate analysis of variance (PERMANOVA) based on the robust Aitchison dissimilarity distance [49]. The relative effects of ‘time’, ‘treatment’, and ‘time × treatment’ were tested using the *adonis2* function in the *vegan* package [47] with 999 permutations, and a significance threshold *p* < 0.001; the “strata” option was used to constrain the permutations of samples within each time point. The abundance of soil prokaryotic and fungal phyla was subjected to a differential abundance analysis using the DESeq2 method to compare the cumulative effect of digestate to the other treatments [50].

Finally, a variance partitioning approach was used to determine if the amounts of organic carbon and fertilizing elements (NPK) applied over 10 years could explain the variability observed in the dynamics of the soil molecular microbial biomass and the soil microbial community structure. For this purpose, the most parsimonious additive model was selected according to a forward selection procedure by minimizing the Akaike information criterion (AIC), using the *rda* and *ordistep* functions in the *vegan* package [47].

## 3 Results

### 3.1 OW and soil chemical characteristics

Over a decade of repeated application of different organic wastes (OW), no statistically significant differences were observed between the physicochemical properties of undigested (PS) and digested (PS-DIG) organic matter (*p* > 0.05, Table 1). In contrast, cattle manure (CM) exhibited significantly higher concentrations of carbon and nitrogen (*p* < 0.05, Table 1). In 2012, before any treatment application, the physicochemical properties of the soil were similar in all plots (*p* > 0.05, Table 2). Similarly, in 2016, no statistically significant differences were observed between treatments (*p* > 0.05, Table 2). After a decade, in 2022, the soil chemical properties were lastingly modified, and the magnitude of the changes was treatment-dependent. Specifically, the soil organic carbon (SOC) content significantly decreased by an average of 7 % in the plots treated with PS and PS-DIG, and by 14 % in the MIN and ON plots (*p* < 0.05, Table S 2). The only plots that maintained a stable SOC were those amended with CM (*p* > 0.05, Table 2 and Table S 2). All treatments resulted in a significant decrease in the C/N ratio over time, by 7 % on average (*p* < 0.05, Table S 2). Significant soil acidification was observed in the MIN plots (*p* < 0.05, Table 2 and Table S 2), while the soil pH remained stable over time in the ON, PS, and PS-DIG plots (*p* > 0.05, Table 2 and Table S 2). In contrast, the pH significantly increased in the CM plots (*p* < 0.05, Table 2 and Table S 2).

### 3.2 Effects of organic and inorganic fertilization on soil molecular microbial biomass

**Fig. 2:**
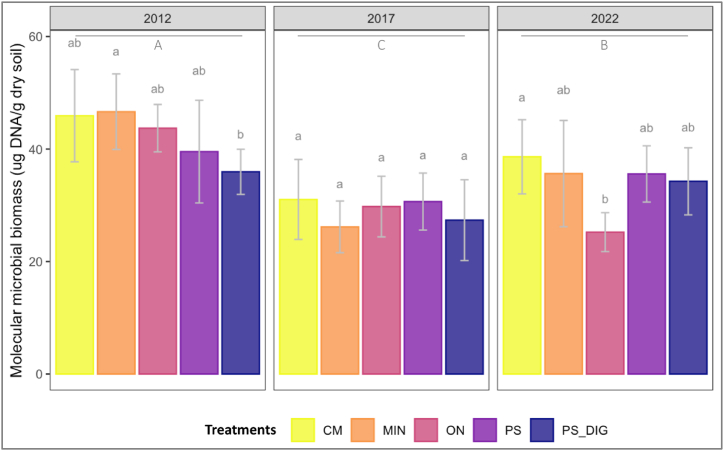
Soil molecular microbial biomass dynamics by year at the EFELE (experimental field site in Rennes, France) from 2012 to 2022. Treatments: MIN, mineral fertilizer; ON, control that received no organic or mineral N input; three different OW: CM, cattle manure; PS, pig slurry; PS-DIG, pig slurry digestate. Capital letters, differences among sampling time; lower-case letters, effect of the treatment for each sampling time. Different letters indicate significant differences (*p* < 0.05) based on Tukey's HSD test; *p*-value adjusted by the BH method.

A significant ‘year’ effect was observed on soil molecular microbial biomass regardless of the treatment (*p* < 0.05, Fig. 2). Overall, across all plots, soil molecular microbial biomass decreased by 32 % in 2017 compared to the beginning of the trial (2012) (*p* < 0.05, Fig. 2). In 2012, before any fertilizer application, a heterogeneity in soil molecular microbial biomass was observed, particularly between plots selected for mineral fertilization treatment and those designated to receive digestate (*p* < 0.05, Fig. 2; on average, 23 % less biomass in PS-DIG plots compared to MIN plots). After ten years (in 2022), significant differences were observed between plots amended with cattle manure (CM) and those unfertilized control plots (ON) (Fig. 2), where the highest soil molecular microbial biomass was recorded when CM was repeatedly applied over a decade and lowest in ON plots (*p* < 0.05, Fig. 2; on average 35 % more biomass in CM plots compared to ON plots). The redundancy analysis (RDA) model identified the parameters that explained variations in soil molecular microbial biomass over a decade (Table 3). The temporal factor (‘year’) strongly influenced the model, explaining 43.3 % of the variance. In contrast, cumulative C organic inputs explained 4.2 % of the variation in soil molecular microbial biomass.

**Table 3:** Effects of the cumulative inputs during the decade (2012–2022) at the EFELE site on the soil molecular microbial biomass and microbial community structure.

Parameter	Explanatory variables	*F*	*P*-value	Variance explained (%)
**Soil molecular microbial biomass**	Year	23.09	0.001	43.3
	C organic inputs	4.49	0.041	4.2
	Residual	–	–	52.5
**Structure of soil Prokaryotic community**	Year	8.14	0.001	21.3
	C/N inputs	8.99	0.001	11.8
	Residual	–	–	66.8
**Structure of soil Fungal community**	Year	50.98	0.01	64.1
	Residual	–	–	35.9

Variance partitioning approach, using Redundancy Analysis (RDA) to select the most parsimonious additive model, according to a forward selection procedure. F (Fisher's F-value), and p-value of Fisher's F from ANOVA test of RDA selected model. Scores from the NMDS ordination based on the robust Aitchison dissimilarity distance were employed for the variance partitioning approach of the soil microbial community structure.

### 3.3 Effects on microbial diversity and community structure

**Fig. 3:**
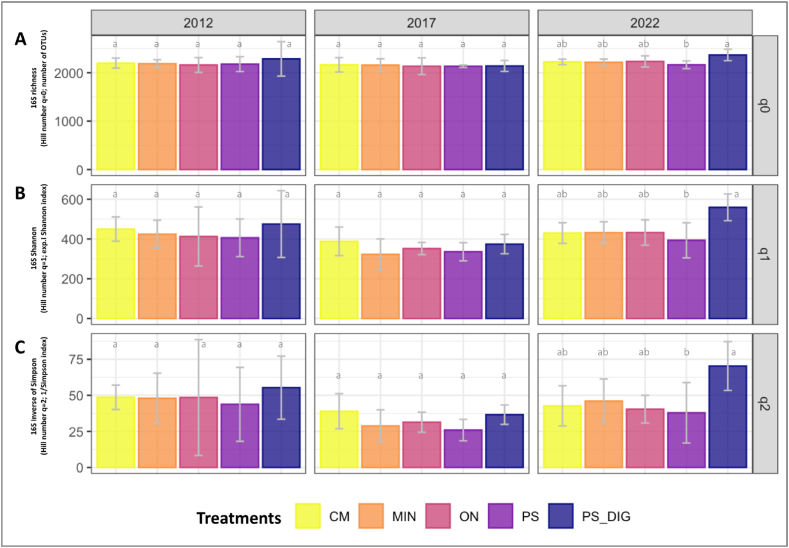
Soil prokaryotic community diversity dynamics by year at the EFELE (experimental field site in Rennes, France) based on Hill numbers. Hill numbers allow interpreting alpha diversity linearly by progressively considering the abundances (q-values) of rare and dominant OTUs. Therefore, (**A**) q0 represents richness, (**B**) q1 is the exponential of Shannon entropy, and (**C**) q2 is the inverse of Simpson index. Treatments: MIN, mineral fertilizer; ON, control that received no organic or mineral N input; three different OW: CM, cattle manure; PS, pig slurry; PS-DIG, pig slurry digestate. Lower-case letters: effect of the treatment for each sampling time. Different letters indicate significant differences (*p* < 0.05) based on Tukey's HSD test; *p*-value adjusted by the BH method.

**Fig. 4:**
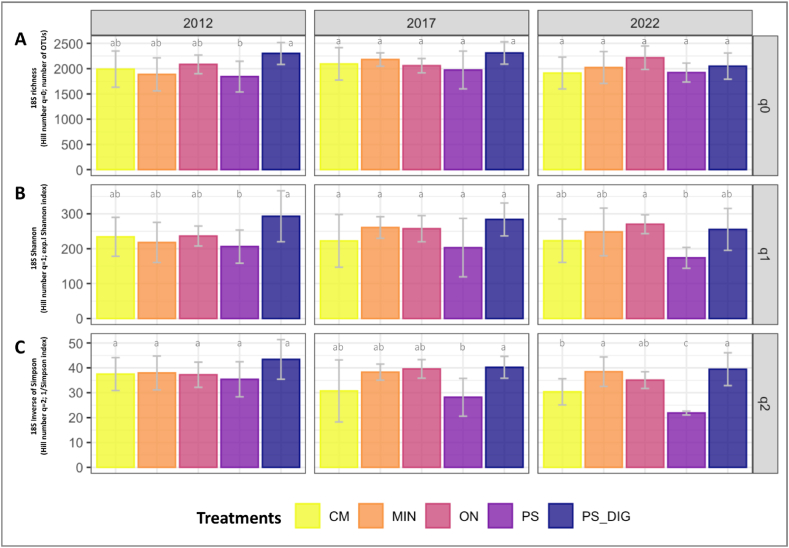
Soil fungal community diversity dynamics by year at the EFELE (experimental field site in Rennes, France) based on Hill numbers. Hill numbers allow interpreting alpha diversity linearly by progressively considering the abundances (q-values) of rare and dominant OTUs. Therefore, (**A**) q0 represents richness, (**B**) q1 is the exponential of Shannon entropy, and (**C**) q2 is the inverse of Simpson index. Treatments: MIN, mineral fertilizer; ON, control that received no organic or mineral N input; three different OW: CM, cattle manure; PS, pig slurry; PS-DIG, pig slurry digestate. Lower-case letters: effect of the treatment for each sampling time. Different letters indicate significant differences (*p* < 0.05) based on Tukey's HSD test; *p*-value adjusted by the BH method.

Following a decade of different fertilization regimes, significant differences in prokaryotic diversity indices were only observed in 2022 between digestate (PS-DIG) and pig slurry (PS) treated plots (*p* < 0.05, Fig. 3). Plots fertilized with digestate displayed higher values of richness (q = 0), OTUs frequency (q = 1), and effective number of dominant OTUs (q = 2) compared to PS plots (Fig. 3). Regarding fungal community diversity indices, at the beginning of the experiment in 2012, significant differences in richness (q = 0) and OTUs frequency (q = 1) were observed, particularly among plots designated to receive pig slurry (PS) and digestate (PS-DIG) (*p* < 0.05, Fig. 4A and. B). Repeated digestate application over a decade induced a higher soil fungal effective number of dominant OTUs (q = 2) than in soils receiving slurry (PS) or cattle manure (CM) (*p* < 0.05; Fig. 4C).

**Fig. 5:**
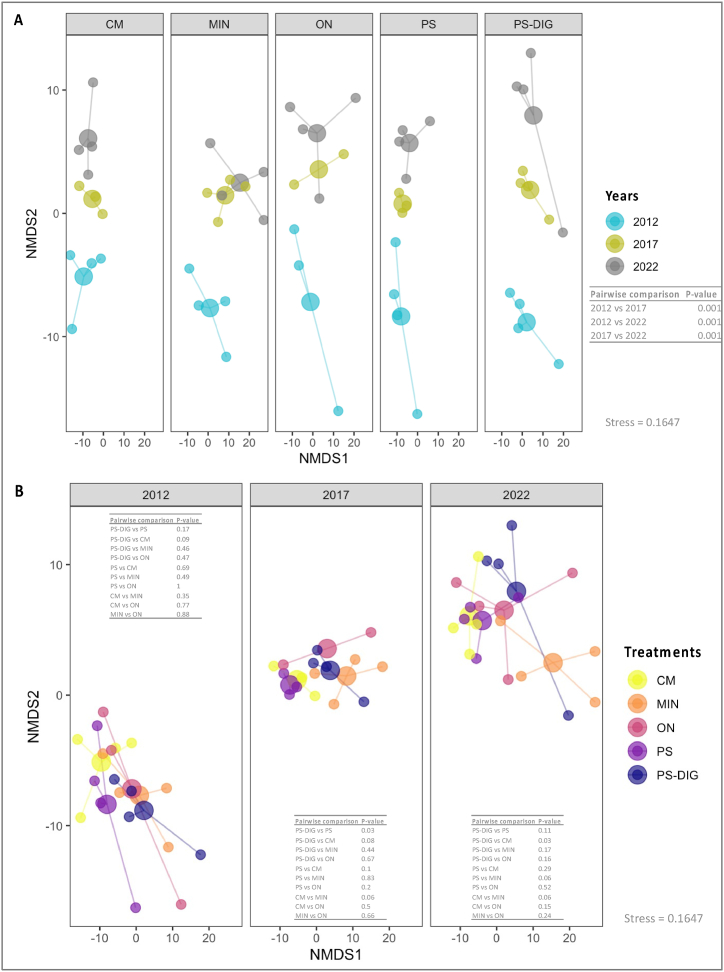
Non-metric multi-dimensional scaling (NMDS) ordination and PERMANOVA pairwise comparisons derived from robust Aitchison dissimilarity distances for (**A, B**) the prokaryotic community at the EFELE (experimental field site in Rennes, France) over 10 years of trial. Treatments: MIN, mineral fertilizer; ON, control that received no organic or mineral N input; three different OW: CM, cattle manure; PS, pig slurry; PS-DIG, pig slurry digestate. Big circles represent centroids, and little circles represent samples. **(A)** prokaryotic community structure colored by year, **(B)** prokaryotic community structure colored by treatment.

**Fig. 6:**
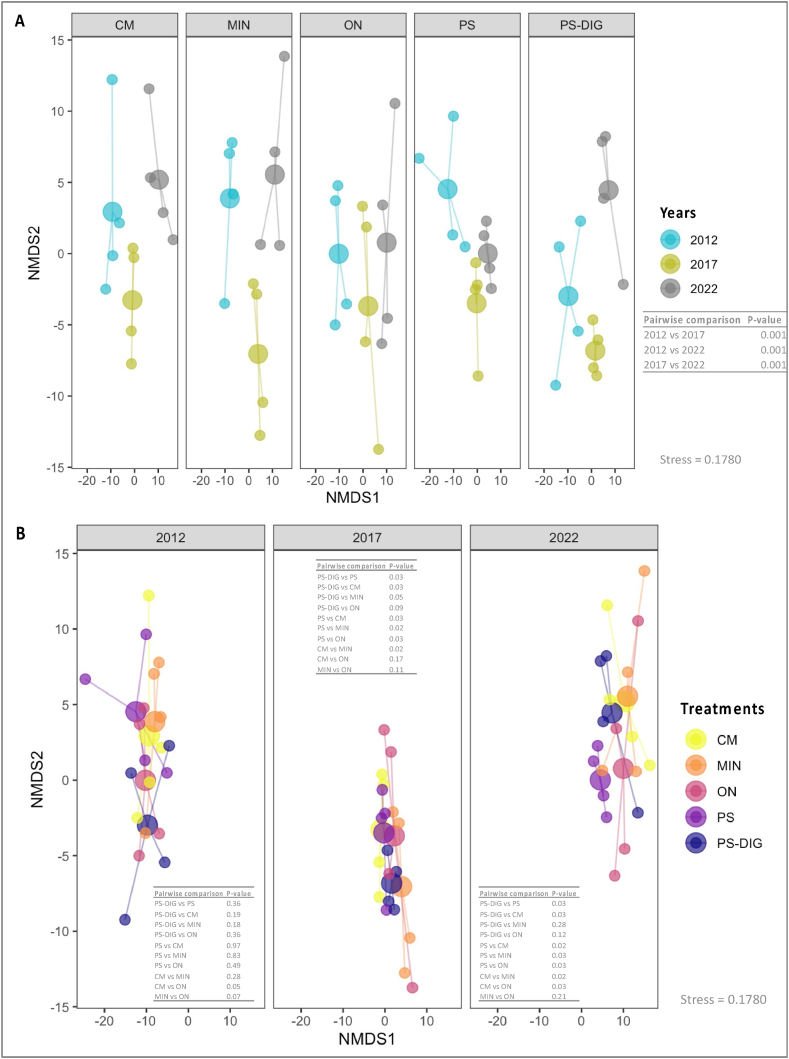
Non-metric multi-dimensional scaling (NMDS) ordination and PERMANOVA pairwise comparisons derived from robust Aitchison dissimilarity distances for (**A, B**) the fungal community at the EFELE (experimental field site in Rennes, France) over 10 years of trial. Treatments: MIN, mineral fertilizer; ON, control that received no organic or mineral N input; three different OW: CM, cattle manure; PS, pig slurry; PS-DIG, pig slurry digestate. Big circles represent centroids, and little circles represent samples. **(A)** fungal community structure colored by year, and **(B)** fungal community structure colored by treatment.

The analysis of similarities revealed significant differences in the microbial community structure (prokaryotic and fungal) among years and treatments (ANOSIM; *p* < 0.05, Table 4), although the ‘year’ effect seemed greater than the ‘treatment’ effect (ANOSIM; R statistic, Table 4). The analysis of multivariate homogeneity of group dispersions demonstrated that the dispersion of community structure differs significantly between years but not between treatments (beta-dispersion; Table 4). NMDS ordination confirmed these findings (Fig. 5, Fig. 6), which clearly highlighted distinct clustering by ‘year’ (Fig. 5, Fig. 6A), indicating that the changes of soil microbial communities were more influenced by time than by fertilization type. PERMANOVA confirmed that both ‘year’ and ‘fertilization type’ had a significant effect on the prokaryotic and fungal community structure (*p* < 0.05, Table 5). Pairwise comparisons revealed lasting modifications over time, with the largest differences between 2012 and 2022 (Fig. 5, Fig. 6A). After 10 years, the microbial community structure in the plots receiving digestate (PS-DIG) differed significantly from those amended with cattle manure (both prokaryotic and fungal community structure) and pig slurry (fungal community structure) (Fig. 5, Fig. 6B). In contrast, no significant differences were observed when comparing the microbial community structures between digestate fertilized plots and mineral fertilized (MIN) or unfertilized ones (ON) (Fig. 5, Fig. 6B).

**Table 4:** Analysis of similarities (ANOSIM), and analysis of multivariate homogeneity of group dispersions (beta-dispersion) depicting the differences in the prokaryotic and fungal community compositions between and within groups (‘treatment’ and ‘time’) based on the Robust-Aitchison distance matrices at the EFELE (experimental field site in Rennes, France), according to the treatment over time (999 permutations, significance threshold *p* < 0.05).

	Prokaryotic community			Fungal community		
	ANOSIM		Beta-dispersion	ANOSIM		Beta-dispersion
	R statistic	*P*-value	*P*-value	R statistic	*P*-value	*P*-value
**Treatment**	0.14	0.002	0.24	0.13	0.002	0.88
**Year**	0.29	0.001	0.04	0.60	0.001	0.01

R statistic: degree de separation between test groups ranging from −1 to 1; R = 0, not different; R = 1, completely different. Significance values were based on 999 permutations.

**Table 5:** Permutational multivariate analysis of variance **(**PERMANOVA) results depicting the differences in the prokaryotic and fungal community compositions based on the robust Aitchison distance matrices, at the EFELE (experimental field site in Rennes, France), according to the treatment over time (999 permutations, significance threshold *p* < 0.05).

	Prokaryotic community				Fungal community			
	SS	R^2^	*F*	*P*-value	SS	R^2^	*F*	*P*-value
**Treatment**	0.44	0.11	1.78	0.001	0.69	0.07	2.88	0.001
**Year**	0.47	0.12	3.83	0.02	1.79	0.19	5.92	0.001
**Treatment × Year**	0.43	0.11	0.88	0.87	1.53	0.16	1.26	0.001
**Residual**	2.51	0.65	–	–	5.44	0.57	–	–
**Total**	3.84	1	–	–	9.46	1	–	–

SS (Sum of Squares), R^2^ (Determination coefficient), F (Fisher's F-value), and p-value of Fisher's.

The redundancy analysis (RDA) model identified the parameters that explain variations in the microbial community structure over a decade (Table 3). The temporal factor (‘year’) had the strongest influence in both models. It represented 21.3 % and 64.1 % of the explained variance of the prokaryotic and fungal community structure, respectively (Table 3). The C/N ratio of the applied product represented 11.8 % of the explained variance of the soil prokaryotic community structure. In contrast, none of the chemical properties of the treatments applied (cumulative organic carbon or NPK fertilizing elements) were able to explain the dynamics of variation in the structure of soil fungal communities (Table 3).

**Fig. 7:**
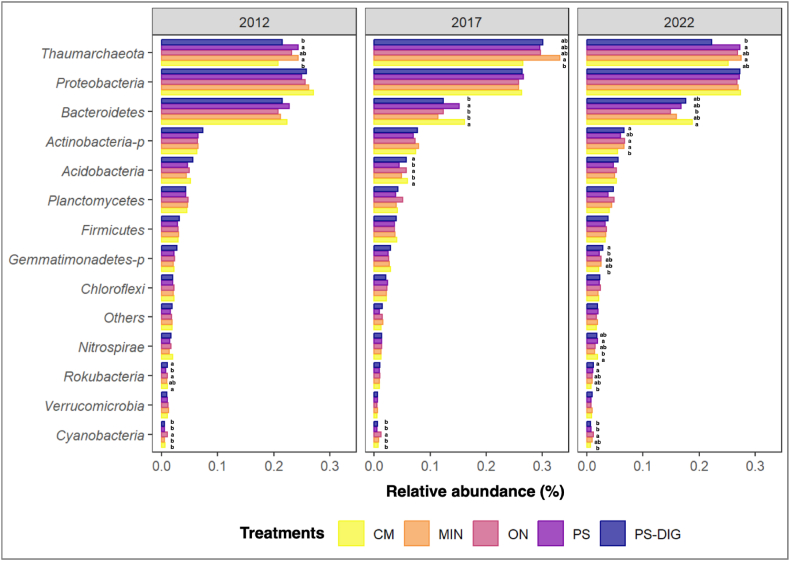
Relative abundances of the major prokaryotic phyla by year at the EFELE (experimental field site in Rennes, France) from 2012 to 2022. Treatments: MIN, mineral fertilizer; ON, control that received no organic or mineral N input; three different OW: CM, cattle manure; PS, pig slurry; PS-DIG, pig slurry digestate. Relative taxonomic abundances below 5 % were grouped in “Others”. Lower-case letters, effect of the treatment for each sampling time. Different letters indicate significant differences (*p* < 0.05) based on Tukey's HSD test; *p*-value adjusted by the BH method.

**Fig. 8:**
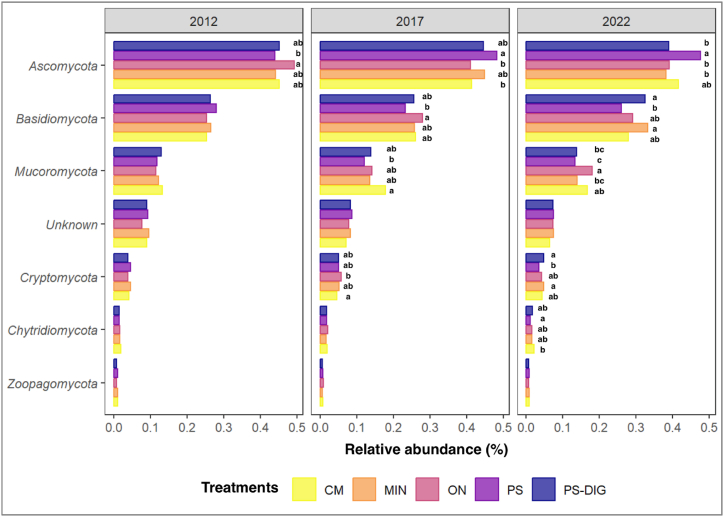
Relative abundances of the major fungal phyla by year at the EFELE (experimental field site in Rennes, France) from 2012 to 2022. Treatments: MIN, mineral fertilizer; ON, control that received no organic or mineral N input; three different OW: CM, cattle manure; PS, pig slurry; PS-DIG, pig slurry digestate. Lower-case letters, effect of the treatment for each sampling time. Different letters indicate significant differences (*p* < 0.05) based on Tukey's HSD test; *p*-value adjusted by the BH method.

The temporal changes in the relative abundances of dominant soil microbial phyla over a decade following different fertilization regimes are illustrated in Fig. 7, Fig. 8. Overall, 13 prokaryotic and 6 fungal major phyla were identified across all treatments and years considered. The prokaryotic phyla were *Acidobacteria, Actinobacteria-p, Bacteroidetes, Chloroflexi, Cyanobacteria, Firmicutes, Gemmatimonadetes-p, Nitrospirae, Planctomycetes, Proteobacteria, Rokubacteria*, *Thaumarchaeota*, and *Verrucomicrobia*. The fungal phyla were *Ascomycota, Basidiomycota, Chytridiomycota, Cryptomycota, Mucoromycota*, and *Zoopagomycota*. When comparing digestate to other OW, the repeated digestate application over a decade induced lower relative abundance in *Thaumarchaeota* but higher in *Actinobacteria-p, Gemmatimonadetes-p,* and *Rokubacteria* (Fig. 7). For the fungal phyla, digestate favored *Basidiomycota* and *Cryptomycota* (Fig. 8). The differential abundance analysis conducted using the DESeq2 method provided insights into the changes in the abundance of various microbial phyla in response to different fertilization treatments. Notably, when comparing the cumulative effect of a decade of annual digestate application to other treatments, fewer significant differences at the phylum level were observed between PS-DIG and MIN-treated plots, specifically in the Archaeal phyla *Thaumarchaeota* and *Euryarchaeota*, and the fungal phylum *Blastocladiomycota* (Fig. S 4 and Fig. S 5; *p* < 0.05).

## 4 Discussion

Comparing to laboratory approaches, employing an *in-situ* methodology in real agronomical and pedological conditions provides a more comprehensive understanding of short- (≤ 1 year), mid- (≤ 5 years), and long- (≥ 6 years) term impact of digestates on soil microbiota. To our knowledge, no prior study has employed an *in-situ* approach to monitor the effects of repeated digestate applications over a decade. The overall objective of this study was to assess the long-term effects of repeated digestate applications as an organic fertilizer on soil microbial parameters, including molecular microbial biomass, diversity, structure, and composition. Furthermore, we aimed to evaluate how these effects relate to changes in edaphic soil properties, hypothesizing that significant modifications would emerge only after several years of repeated applications.

### 4.1 Cumulative effects of fertilization treatment on soil edaphic properties

As previously observed with a liquid digestate by Ref. [51], the repeated application of digestate did not induce a lasting increase of SOC content as commonly observed with classical OW such as farmyard manure or compost [52]. The stabilization or increase of the SOC content was observed depending on the OW type and quantity applied. In the present study, CM plots resulted in a stable SOC content throughout the 10 years of the experiment, primarily attributed to the large quantity of organic carbon applied (total organic C (TOC) in the CM treatment: 28,155 kg ha^_1^), significantly higher than the other treatments. In contrast, a lower quantity of organic carbon was applied *via* digestate and pig slurry (TOC input: 8445 kg ha^_1^ in the PS plots, 6453 kg ha^_1^ in the PS-DIG plots), explaining why the SOC content could not be maintained throughout the years. These findings align with the literature, emphasizing that increasing total C input is the primary driver for maintaining or rising SOC stocks [53]. Moreover, as previously reported for organic farming systems, rising SOC levels is a gradual process closely related to biological soil quality and the quality of inputs materials [54]. The treatments induced distinct dynamics of the total soil nitrogen content. These variations were attributed to differences in the proportions of total N applied *via* each treatment; specifically, the plots fertilized with digestate and mineral fertilizer recorded lower total soil N content at the end of the experiment. Indeed, over a decade of repeated fertilization, the cumulative total nitrogen supplied was lower in plots fertilized with digestate or mineral fertilizer compared to plots amended with organic products (cattle manure and pig slurry) (CM plots 2280 kg ha^−1^; PS plots 1606 kg ha^−1^; PS-DIG plots 1594 kg ha^−1^; and MIN plots 1109 kg ha^−1^). In line with prior studies, differences in carbon and nitrogen contents in the OW led to high variability in the C/N ratios across treatments [55]. The C/N ratio of the OW is an indicator of their nitrogen mineralization capacity. When OW with C/N ratios below 20 are added to soils, net mineralization dominates [56]. The C/N ratios below 10 favor rapid organic matter mineralization, while values above 10 result in slower mineralization [57]; microbial N immobilization becomes the dominant process with higher C/N ratios [58]. An optimal C/N ratio in the range of 5–6 has been proposed for OW [56,59]. In the present study, the C/N ratios of digestate ranged between 2.9 and 5.8, and for the other OW (pig slurry and cattle manure), the values ranged between 3.5 and 8.3 (PS) and 12.8 and 19 (CM), respectively. The lower C/N ratios of digestate may also explain why these inputs did not contribute to increases in the SOC content, favoring rapid mineralization of the organic matter and potentially resulting in different fates for nitrogen in the amended soils [55]. Regarding soil pH, our study found no significant changes after 10 years of repeated digestate application, consistent with previous *in-situ* studies that also reported stable soil pH levels following repeated digestate inputs [51,[60], [61], [62]].

### 4.2 Cumulative effect of digestate application on soil molecular microbial biomass

During the biogas production process, anaerobic microorganisms degrade most of the readily available carbon, leading to the subsequent production of methane and carbon dioxide [63]. Consequently, a reduced amount of readily available carbon for soil microorganisms, and therefore a lower microbial biomass, can be expected when digested organic matter is used as an amendment compared to undigested organic matter [64]. Contrary to these expectations, our findings highlighted that the soil molecular microbial biomass content was similar in plots treated with OW (digestate (PS-DIG), undigested organic matter (PS), and cattle manure (CM)), although their SOC content differed. As reported by Ref. [51], increases in SOC content were associated with improvements in soil biological activity and particularly in microbial biomass. Moreover, higher soil microbial biomass has been observed in plots amended with cattle manure [26,54,65]. The results obtained in our study could be explained by a buffering effect of agricultural practices (e.g., crop rotation, the reasoning of the input doses, cover and catch crop) which exert a compensatory effect on soil molecular microbial biomass, not allowing for the observation of a more pronounced treatment-effect. Indeed, it is known that certain practices, such as the diversity of crop rotations [66] or soil tillage [67], can impact soil microbial biomass. In addition, the variance partitioning approach identified the temporal parameter (‘year’) as the main factor explaining variations in soil molecular microbial biomass, with a significant and systematic decrease in biomass in all treatments in 2017. The winter of 2016/17 was drier and colder than usual [68]. Typically, drainage seasons commenced between November and January, except for the winter of 2016/17, which started in February, lasted only one month, and had notably lower rainfall, resulting in reduced drainage volumes [68]. This underscores the crucial role of soil temperature and moisture in driving seasonal changes in soil microbial communities. By influencing temperature and precipitation, climate can either mitigate or exacerbate the impact of land use on the soil microbial biomass [69]. Moreover, the season of soil sampling may exert a greater influence on soil microbial communities than the application of digestate [19]. Thus, in our study, land use and soil management practices probably buffered the effects of the treatments, whether organic or mineral, on the soil microbial biomass. These findings highlight the importance of fertilization and soil amendment, and the critical need for a long-term assessment of the potential impacts of any agricultural practice on soil microorganisms.

### 4.3 The historical and cumulative fertilization regime drives the structure of the soil microbial communities

As observed in previous studies, our results highlighted that repeated application of different fertilization treatments induced minor significant differences over time in alpha diversity indices (richness, the exponential of Shannon entropy, and the inverse of Simpson index) [52,70]. Our findings highlighted that the long-term fertilization with digestate stimulated dominant OTUs more than other OW (pig slurry and cattle manure). Furthermore, our results revealed shifts and lasting modifications of the prokaryotic and fungal community structures in relation to time and the fertilization treatment, consistent with prior research, yearly organic and mineral fertilization treatments had a significant long-term impact on the soil microbial community structure [26,70]. Field experiments have shown no significant short-term changes in the soil microbial community structure following digestate application [19,71]. This indicates a different response of the microbial community structure to short- and long-term nutrient additions, whose effects take time before they become noticeable. Moreover, our results highlighted that (i) at least 5 years went by before lasting modifications of the soil microbial community structure were detected, and (ii) the distribution of the prokaryotic structure of the digestate fertilized plots was in between those of the mineral fertilizer and the other OW (pig slurry (PS) and cattle manure (CM)).

The variance partitioning approach revealed that the temporal factor (‘year’) and, to a lesser extent, the cumulative C/N inputs best explained variations in the microbial community structure. As observed for the molecular microbial biomass, these findings underscored that the ‘year’ factor exerted a strong influence, likely reflecting environmental, climatic, and seasonal effects that could mask the impact of repeated applications of different fertilization treatments on soil microorganisms. Nevertheless, the nature of the inputs also plays a significant role in shaping these microbial communities. These findings align with previous studies which emphasize that both mineral and organic long-term fertilization can lead to changes in the soil microbial community composition [28,72,73], due to the stimulation of soil-borne microbes responding to the persistent modifications of the soil physicochemical properties resulting from the chemical composition of the applied OW [74,75].

In our study, the variations of the soil microbial community composition were further reflected by the distinct proportions of the fungal and prokaryotic groups. The most abundant taxa were *Proteobacteria* (∼27 %)*, Thaumarchaeota* (∼25 %)*, Bacteroidetes* (∼19 %), and *Actinobacteria-p* (∼6.4 %). These are the major bacterial and archaeal taxa found in French soils [76]. A decade of repeated digestate application resulted in a lower abundance of *Thaumarchaeota* and a higher abundance of *Actinobacteria-p* compared to the other OW (pig slurry and cattle manure). The phylum *Thaumarchaeota* comprises dominant archaea in the soil prokaryotic community, known for their pivotal role in soil ammonia oxidation by converting ammonia to nitrite and, subsequently, nitric oxide [77]. Thus, a higher abundance of *Thaumarchaeota* could be expected after ammonia inputs *via* the digestate. However, contrary to these expectations, an antagonistic effect may be revealed as previously reported that oxygen availability might be suboptimal for soil-aerobic *Thaumarchaeota* [77]; therefore, our results could suggest a potential long-term consequence of the repeated application of an anaerobic digested product on the abundance of this phylum. However, to date, the impact of digestate application on the soil *Thaumarchaeota* phylum remains poorly documented. The phylum *Actinobacteria* is a Gram-positive, saprophytic, ubiquitous bacteria. They can produce a wide array of extracellular hydrolytic enzymes that degrade complex macromolecules that can break down animal and plant biomass. This makes them central organisms in carbon recycling [2,78]. They are considered as oligotrophs because they dominate the decomposition of organic material when nutrients are limited [79]. An increase in bacteria belonging to this phylum has already been observed after 42 days of digestate application [55]. Furthermore, members of the *Actinobacteria* phylum have been reported as potassium-solubilizing and/or phosphate-solubilizing bacteria [78]. The observed changes in the relative abundance of *Actinobacteria-p* may be linked to the cycling of phosphorus in the soil because the phosphorus content of the OW is one of the limiting elements to determine the application rates at the EFELE site.

The most abundant fungal phyla in our *in-situ* approach were *Ascomycota* (∼43 %)*, Basidiomycota* (∼25 %)*, Mucoromycota* (∼16 %), and “Unknown Fungi” (∼8 %). These are worldwide dominant taxa in soil fungal communities [80]. *Ascomycota* and *Basidiomycota* are major contributors to soil carbon cycling [7]. *Ascomycota* members are generally defined as copiotrophic saprophytes. They are typically characterized as fast-growing, effective decomposers of labile carbon and are abundant dwellers of soils with high C availability [81]. By contrast, *Basidiomycota* members are defined as oligotrophic saprophytes and symbiotrophs [81]. *Basidiomycota* are assumed to possess improved metabolic capacities over *Ascomycota* for decomposing more recalcitrant soil C compounds [7]. In 2022, the higher abundances of *Ascomycota* in plots amended with traditional organic products (PS and CM) may be attributed to organic carbon availability within these treatments. In contrast, the higher relative abundance of *Basidiomycota* in plots receiving digestate or mineral fertilizer may be explained by the reduced amount of readily available carbon for soil microorganisms when digested organic matter is used as an amendment, having a similar effect to a mineral fertilizer [29].

## 5 Conclusions

This study provides the first field-based analysis of the dynamic effects of repeated digestate applications over a decade, assessing whether the impacts of digestate differ from those of its undigested feedstock and other commonly used fertilizers. Our findings provided new insights regarding the long-term effects of one type of digestate on soil microorganisms. After a decade of repeated applications of different OW (cattle manure, pig slurry and digestate of pig slurry) or mineral fertilizer, lasting changes were observed in the soil's physicochemical properties and microbial parameters across all treatments. These changes were primarily explained by the effect of time and to a lesser extent by the quality of the applied product. Our results highlight that the response of soil microbial communities to repeated digestate application is similar to that of mineral fertilizer but strongly influenced and buffered by other agricultural practices and environmental and climatic conditions. Hence, when assessing the impact of digestate on soil microbial communities, associated agricultural practices should be considered. Further research is needed to better understand the cumulative effects of repeated digestate applications on soil microbial communities, particularly across diverse agro-pedological contexts. This includes not only soil type but also interactions with agricultural practices, such as fertilization history, additive fertilization (additional nutrient inputs), tillage, and plant cover (e.g., crop type, cover crops, rotation, and plant diversity).

## CRediT authorship contribution statement

**Daniela Mora-Salguero:** Writing – original draft, Methodology, Formal analysis. **Lionel Ranjard:** Writing – review & editing. **Thierry Morvan:** Writing – review & editing, Methodology. **Samuel Dequiedt:** Writing – review & editing, Formal analysis, Data curation. **Vincent Jean-Baptiste:** Writing – review & editing. **Sophie Sadet-Bourgeteau:** Writing – review & editing, Supervision, Project administration, Funding acquisition, Formal analysis, Conceptualization.

## Funding

This project was funded by Institut Agro Dijon. The funders had no role in the study design, data collection and analysis, decision to publish, or preparation of the manuscript.

## Declaration of competing interest

The authors declare that they have no known competing financial interest or personal relationships that could have appeared to influence the work reported in this paper.

## Footnotes

1 https://valor-pro.hub.inrae.fr/.

2 https://www2.dijon.inrae.fr/plateforme_genosol/.

3 https://www6.hautsdefrance.inrae.fr/las.
