# Supplementary material for: Long-term effect of repeated application of pig slurry digestate on microbial communities in arable soils
Source: Heliyon. 2024 Dec 10;11(1):e41117. doi: 10.1016/j.heliyon.2024.e41117 (PMC11699314; doi:10.1016/j.heliyon.2024.e41117)
Supplement: Multimedia component 1 [file mmc1.docx]

## Supplementary material

Table S 1. Amountsof organic carbon and fertilizing elements (NPK) applied *per* year from 2012 to 2021 at the EFELE (experimental field site in Rennes, France). Treatments: MIN, mineral fertilizer; ON, control that received no organic or mineral N input; three different OW: CM, cattle manure; PS, pig slurry; PS-DIG, pig slurry digestate. For the OW, mineral N represents the amount of ammoniacal N (NH_4_^+^).

| **Treatment** | **Year** | **Mineral N** | **Organic N** | **Total N** | **Organic C** | **K_2_O** | **P_2_O_5_** |
| --- | --- | --- | --- | --- | --- | --- | --- |
|  |  | **kg ha^-1^** | | | | | |
| CM | 2012 | 100.01 | 206.46 | 306.47 | 4055.81 | 797.13 | 164.67 |
| MIN | 2012 | 80.00 | 0.00 | 80.00 | 0.00 | 60.00 | 50.00 |
| ON | 2012 | 0.00 | 0.00 | 0.00 | 0.00 | 60.00 | 50.00 |
| PS | 2012 | 106.24 | 37.44 | 143.68 | 724.46 | 189.60 | 67.58 |
| PS_DIG | 2012 | 104.14 | 31.35 | 135.49 | 445.40 | 185.54 | 65.32 |
| CM | 2013 | 116.00 | 0.00 | 116.00 | 0.00 | 0.00 | 0.00 |
| MIN | 2013 | 116.00 | 0.00 | 116.00 | 0.00 | 0.00 | 0.00 |
| ON | 2013 | 0.00 | 0.00 | 0.00 | 0.00 | 0.00 | 0.00 |
| PS | 2013 | 179.52 | 67.39 | 246.91 | 941.15 | 424.76 | 148.66 |
| PS_DIG | 2013 | 189.34 | 53.57 | 242.91 | 918.15 | 467.75 | 173.11 |
| CM | 2014 | 27.43 | 232.75 | 260.18 | 4956.76 | 1151.75 | 154.74 |
| MIN | 2014 | 102.00 | 0.00 | 102.00 | 0.00 | 0.00 | 0.00 |
| ON | 2014 | 0.00 | 0.00 | 0.00 | 0.00 | 0.00 | 0.00 |
| PS | 2014 | 114.49 | 29.98 | 144.47 | 518.56 | 304.10 | 99.05 |
| PS_DIG | 2014 | 132.12 | 41.50 | 173.62 | 556.05 | 333.37 | 77.50 |
| CM | 2015 | 120.00 | 0.00 | 120.00 | 0.00 | 0.00 | 0.00 |
| MIN | 2015 | 120.00 | 0.00 | 120.00 | 0.00 | 0.00 | 0.00 |
| ON | 2015 | 0.00 | 0.00 | 0.00 | 0.00 | 0.00 | 0.00 |
| PS | 2015 | 159.53 | 47.06 | 206.59 | 1023.08 | 345.19 | 126.84 |
| PS_DIG | 2015 | 135.24 | 38.23 | 173.48 | 694.27 | 564.27 | 161.86 |
| CM | 2016 | 68.00 | 200.50 | 268.50 | 4649.30 | 1170.22 | 159.06 |
| MIN | 2016 | 96.00 | 0.00 | 96.00 | 0.00 | 120.00 | 100.00 |
| ON | 2016 | 0.00 | 0.00 | 0.00 | 0.00 | 120.00 | 100.00 |
| PS | 2016 | 98.28 | 39.78 | 138.06 | 723.74 | 218.73 | 85.45 |
| PS_DIG | 2016 | 92.40 | 31.20 | 123.60 | 361.44 | 124.81 | 71.69 |
| CM | 2017 | 61.00 | 0.00 | 61.00 | 0.00 | 0.00 | 0.00 |
| MIN | 2017 | 80.00 | 0.00 | 80.00 | 0.00 | 120.00 | 0.00 |
| ON | 2017 | 0.00 | 0.00 | 0.00 | 0.00 | 120.00 | 0.00 |
| PS | 2017 | 89.37 | 33.73 | 123.11 | 796.07 | 188.88 | 71.97 |
| PS_DIG | 2017 | 110.69 | 34.92 | 145.62 | 641.40 | 248.13 | 109.59 |
| CM | 2018 | 51.50 | 252.50 | 304.00 | 3897.41 | 1147.93 | 138.78 |
| MIN | 2018 | 126.00 | 0.00 | 126.00 | 0.00 | 120.00 | 100.00 |
| ON | 2018 | 0.00 | 0.00 | 0.00 | 0.00 | 120.00 | 100.00 |
| PS | 2018 | 101.55 | 29.89 | 131.44 | 684.33 | 175.89 | 75.55 |
| PS_DIG | 2018 | 103.00 | 26.18 | 129.18 | 414.49 | 155.56 | 70.56 |
| CM | 2019 | 104.00 | 0.00 | 104.00 | 0.00 | 0.00 | 0.00 |
| MIN | 2019 | 145.00 | 0.00 | 145.00 | 0.00 | 120.00 | 75.00 |
| ON | 2019 | 0.00 | 0.00 | 0.00 | 0.00 | 120.00 | 75.00 |
| PS | 2019 | 91.60 | 28.00 | 119.60 | 616.90 | 242.20 | 103.20 |
| PS_DIG | 2019 | 109.60 | 32.80 | 142.40 | 570.24 | 232.72 | 81.73 |
| CM | 2020 | 76.34 | 234.10 | 310.45 | 4429.02 | 1234.80 | 373.81 |
| MIN | 2020 | 120.00 | 0.00 | 120.00 | 0.00 | 120.00 | 100.00 |
| ON | 2020 | 0.00 | 0.00 | 0.00 | 0.00 | 120.00 | 100.00 |
| PS | 2020 | 101.35 | 25.36 | 126.72 | 536.95 | 179.57 | 175.66 |
| PS_DIG | 2020 | 121.50 | 31.49 | 152.99 | 884.41 | 328.82 | 237.56 |
| CM | 2021 | 67.50 | 362.50 | 430.00 | 6167.01 | 2068.12 | 272.16 |
| MIN | 2021 | 124.00 | 0.00 | 124.00 | 0.00 | 120.00 | 100.00 |
| ON | 2021 | 0.00 | 0.00 | 0.00 | 0.00 | 120.00 | 100.00 |
| PS | 2021 | 148.01 | 77.90 | 225.91 | 1879.60 | 373.43 | 340.81 |
| PS_DIG | 2021 | 129.15 | 46.33 | 175.48 | 966.92 | 142.40 | 109.61 |

Table S 2. Soil physicochemical parameters of the EFELE plots by fertilization treatment across different sampling times.

| **Treatment** | **Organic carbon** | **Total N** | **P_2_O_5_ (Olsen)** | **C:N** | **pH (water)** | **CEC** |
| --- | --- | --- | --- | --- | --- | --- |
|  | **g kg^-1^** | | |  |  | **cmol+ kg^-1^** |
| **CM** | | | | | | |
| **2012** | 10.78a (0.57) | 1.13a (0.05) | 0.18b (0.02) | 9.54c (0.05) | 6.14a (0.05) | 6.17a (0.21) |
| **2016** | 11.13a (0.36) | 1.19ab (0.02) | 0.14a (0.02) | 9.37b (0.09) | 6.17a (0.11) | 6.64a (0.47) |
| **2021** | 11.27a (0.39) | 1.25b (0.05) | 0.15a (0.02) | 9.03a (0.09) | 6.36b (0.14) | 6.55a (0.28) |
| **MIN** | | | | | | |
| **2012** | 11.45c (0.53) | 1.19c (0.06) | 0.18c (0.03) | 9.62c (0.09) | 5.97b (0.24) | 6.10b (0.65) |
| **2016** | 10.80b (0.54) | 1.16b (0.06) | 0.13a (0.02) | 9.28b (0.04) | 5.92ab (0.20) | 6.02ab (0.64) |
| **2021** | 9.89a (0.24) | 1.11a (0.04) | 0.15b (0.02) | 8.89a (0.18) | 5.85a (0.16) | 5.80a (0.45) |
| **ON** | | | | | | |
| **2012** | 11.17c (0.94) | 1.15b (0.09) | 0.19c (0.03) | 9.72c (0.12) | 6.15a (0.27) | 6.36a (0.55) |
| **2016** | 10.42b (0.84) | 1.12ab (0.08) | 0.14a (0.02) | 9.26b (0.14) | 6.13a (0.19) | 6.29a (0.48) |
| **2021** | 9.61a (0.39) | 1.08a (0.05) | 0.17b (0.03) | 8.93a (0.09) | 6.17a (0.31) | 5.67a (1.21) |
| **PS** | | | | | | |
| **2012** | 10.95b (0.44) | 1.14a (0.04) | 0.19b (0.02) | 9.62c (0.08) | 6.16a (0.21) | 6.45a (0.58) |
| **2016** | 10.95b (0.64) | 1.17a (0.06) | 0.15a (0.02) | 9.38b (0.08) | 6.26a (0.14) | 6.62a (0.51) |
| **2021** | 10.14a (0.33) | 1.15a (0.04) | 0.16a (0.01) | 8.83a (0.07) | 6.14a (0.13) | 6.39a (0.46) |
| **PS-DIG** | | | | | | |
| **2012** | 10.52b (0.88) | 1.10a (0.09) | 0.17b (0.01) | 9.57c (0.05) | 5.99a (0.25) | 5.71a (0.41) |
| **2016** | 10.45b (0.65) | 1.13b (0.07) | 0.14a (0.01) | 9.29b (0.03) | 6.08a (0.16) | 5.92a (0.26) |
| **2021** | 9.79a (0.54) | 1.10ab (0.06) | 0.14a (0.01) | 8.94a (0.05) | 6.07a (0.22) | 5.82a (0.28) |

*Data pertains to air-dried soil. Values are means and letters denote the significant effect of sampling time for each treatment (p < 0.05) based on Tukey’s HSD test p-value adjusted by the BH method. The standard error of the means is indicated in parentheses. CM, cattle manure; PS, pig slurry; MIN, mineral fertilizer; 0N, no fertilization; PS-DIG, pig slurry digestate.*

Figure S 1. Cumulative total inputs applied from 2012 to 2021 at the EFELE (experimental field site in Rennes, France). Treatments: MIN, mineral fertilizer; ON, control that received no organic or mineral N input; three different OW: CM, cattle manure; PS, pig slurry; PS-DIG, pig slurry digestate.

Figure S 2.Amounts of organic carbon and fertilizing elements (NPK) applied *per* year from 2012 to 2021 at the EFELE (experimental field site in Rennes, France). Treatments: MIN, mineral fertilizer; ON, control that received no organic or mineral N input; three different OW: CM, cattle manure; PS, pig slurry; PS-DIG, pig slurry digestate.

Figure S 3.Duplicate of Figure S2, excluding carbon inputs, allowing a clearer visualization of the amounts of fertilizing elements (NPK) applied *per* year from 2012 to 2021 at the EFELE (experimental field site in Rennes, France). Treatments: MIN, mineral fertilizer; ON, control that received no organic or mineral N input; three different OW: CM, cattle manure; PS, pig slurry; PS-DIG, pig slurry digestate.

Figure S 4. Differential abundance analysis at the phylum level of the prokaryotic community in 2022 using the DESeq2 method, showing the relationship between the differential expression value (log2 Fold Change) and the statistical significance (p-value). The cumulative effects after a decade of different fertilization treatments were compared, a positive log2 Fold Change value indicates higher abundance compared to the reference condition (the reference condition was the PS-DIG). Phyla shown in red indicate a significant difference between the compared treatments (*p*< 0.05). (A) CM-treated plots compared to PS-DIG treated plots; (B) MIN-treated plots compared to PS-DIG treated plots; (C) ON-control plots compared to PS-DIG treated plots; and (D) PS-treated plots compared to PS-DIG treated plots.

Figure S 5. Differential abundance analysis at the phylum level of the fungal community in 2022 using the DESeq2 method, showing the relationship between the differential expression value (log2 Fold Change) and the statistical significance (p-value). The cumulative effects after a decade of different fertilization treatments were compared, a positive log2 Fold Change value indicates higher abundance compared to the reference condition (the reference condition was the PS-DIG). Phyla shown in red indicate a significant difference between the compared treatments (*p*< 0.05). (A) CM-treated plots compared to PS-DIG treated plots; (B) MIN-treated plots compared to PS-DIG treated plots; (C) ON-control plots compared to PS-DIG treated plots; and (D) PS-treated plots compared to PS-DIG treated plots.
